# Cytomegalovirus associated transverse myelitis in an immunocompetent host with DNA detection in cerebrospinal fluid; a case report

**DOI:** 10.1186/1756-0500-5-364

**Published:** 2012-07-20

**Authors:** Suneth Karunarathne, Dumitha Govindapala, Yapa Udayakumara, Harshini Fernando

**Affiliations:** 1 National hospital of Sri Lanka, Regent street, Colombo, Sri Lanka

**Keywords:** Transverse myelitis, Cytomegalovirus, Immunocompetent, Cerebrospinal fluid, Polymerase chain reaction, Hyper intensity

## Abstract

**Background:**

Cytomegalovirus associated transverse myelitis among immunocompetent adults has been rarely reported. We report a patient presenting with clinical myelitis followed by previously unreported finding of cytomegalovirus deoxyribonucleic acid in cerebrospinal fluid.

**Case report:**

A forty year old immunocompetent male presented with acute onset progressive bilateral lower limb weakness. His spinal magnetic resonance imaging findings, cerebrospinal fluid analysis and clinical picture were compatible with transverse myelitis. Polymerase chain reaction of the cerebrospinal fluid for cytomegalovirus was positive while other infectious agents were not detected by serology or polymerase chain reaction. He was treated with intravenous ganciclovir with partial clinical response.

**Conclusion:**

Viral genome detection in the cerebrospinal fluid was performed but negative in five out of ten reported cases of cytomegalovirus associated transverse myelitis in the immunocompetent host. In previous cases the inability to isolate the virus in cerebrospinal fluid was considered favouring an immunological mechanism leading to pathogenesis rather than direct viral toxicity but this case is against that theory. This case highlights the fact that Cytomegalovirus should be considered as an aetiological agent in patients with transverse myelitis and that the virus may cause serious infections in immunocompetent host. Therefore this report is of importance to neurologists and physicians in general.

## Background

Cytomegalovirus is member of the herpesviridae family and usually causes asymptomatic infections. It remains latent and may reactivate when the host has poor immune responses leading to life threatening infection. Cytomegalovirus (CMV) is a known cause of severe central nervous system (CNS) infection among immunosuppressed patients especially among those infected with human immunodeficiency virus. CNS infections reported in the immunocompetent hosts due to CMV include encephalitis, meningitis, myelitis and the combinations of any of the above [1]. Although randomized trials are lacking, physicians generally tend to use antiviral treatment including ganciclovir and valganciclovir for severe CMV infections. Use of polymerase chain reaction of CSF for CMV has been reccommended for diagnosis of CNS infection [2].

Acute transverse myelitis is a focal inflammatory disorder of the spinal cord with sensory, motor and autonomic dysfunction [3]. Cytomegalovirus has been described rarely as a aetiological agent in immunocompetent adults presenting with transverse myelitis with only ten cases described previously [4-13]. Infact, any form of severe infection with cytomegalovirus is rare [14]. Out of the ten, only five cases describe abnormalities in the magnetic resonance imaging (MRI) of spinal cord in CMV associated transverse myelitis (Table 1). Two reports describe confirmation of CMV infection with detection of CMV deoxyribonucleic acid (DNA) in the serum by polymerase chain reaction (PCR) while one report describe confirmed CMV antigenaemia. There were no reported cases of CMV PCR positivity in cerebrospinal fluid. We describe a immunocompetent adult who had clinical features, neuroimaging findings and CSF analysis compatible with transverse myelitis where CMV DNA was detected in CSF. 

**Table 1:** The comparison of CSF analysis, MRI appearance, CMV serology and PCR of reported cases of CMV associated transverse myelitis in non-immunocompromised patients

**Case report (year) First author**	**CSF protein mg/l**	**CSF White cells/mm**^**3**^	**Blood serology**	**CSF serology**	**CSF PCR**	**Blood PCR**	**MRI findings**
Our Patient (2011)	300	350 (L-92%)	Negative	Negative	Positive	ND	T1 mild swelling (C2-C5)
							T2 hyperintensity
							No contrast enhancement
1 (2006) Ben Abdelhafidh N	430	15 (L-80%)	Positive	ND	Negative	Negative	T1 hypointensity
							T2 hyperintensity
							No contrast enhancement
2 (2005) Rigamonti A	370	11 (L-85%)	Positive	Positive	Negative	Negative	T1 signal abnormality
							T2 hyperintensity
							Contrast enhancement
3 (2003) Fux CA	480	29 (M-95%)	Positive	Negative	Negative	CMV Antigen positive	Negative
4 (2002) Karacostas D	150	0	Positive	Positive	Negative	Positive	T1 Normal
							T2 hyperintensity
							No contrast enhancement
5 (1999) Giobbia M	1240	3(L-100%)	Positive	negative	Negative	positive	T2 hyperintensity
6 (1995) Baig SM	1650	230(M-95%)	Positive	ND	ND	ND	Negative
7 (1993) Miles C	1700	200(PMN-62%)	Positive	ND	ND	ND	Negative
8 (1993) Tobita M	1020	104	Positive	Positive	ND	ND	T2 hyperintensity
9 (1986) Tyler KL	1400	226 (M-76%)	Positive	ND	ND	ND	ND
10(1976) Kabins S	7200	3200 (PMN-100%)	Positive	ND	ND	ND	ND

ND-not done.

Majority of the case show CSF pleocytosis with lymphocyte predominance and T 2 signal hyper intensity in the MRI.

## Transverse myelitis diagnosis criteria

Bilateral (not necessarily symmetric) sensorimotor and autonomic spinal cord dysfunction

Clearly defined sensory level

Progression to nadir of clinical deficits between 4 hours and 21 days after symptom onset

Demonstration of spinal cord inflammation: cerebrospinal fluid pleocytosis or elevated IgG index, or MRI revealing a gadolinium-enhancing cord lesion

Exclusion of compressive, postradiation, neoplastic, and vascular causes

## Case presentation

A forty year old previously healthy Sri Lankan male presented with acute onset bilateral leg weakness which was progressive over two days following a brief febrile illness of three days. He did not give any history of trauma nor there was any history of tuberculosis. Initially he did not have urinary retention nor there was any sensory symptoms. He did not complain of shortness of breathing.

On examination he was found to have asymmetric flaccid paralysis of bilateral lower limbs with power grade one to two. Reflexes were diminished bilaterally and Babinski sign was equivocal. Examination of optic fundi and cranial nerves were normal. There was no demonstrable sensory level and joint position sense was initially preserved. Upper limbs were neurologically normal. Respiratory movements and sphincter function were preserved. Depending on initial findings a clinical diagnosis of Guillain-Barré syndrome was suspected and he was closely observed for the development of respiratory paralysis with monitoring of forced vital capacity and oxygen saturation.

On the fifth day of onset of symptoms his weakness worsened with additional flaccid paralysis of upper limbs and neck muscles. He was catheterized for acute retention of urine. Patient was urgently transferred to a high dependency unit but he did not develop respiratory paralysis necessitating ventilator support. Later he developed bilateral spastic paraparesis and positive Babinski sign with mild paraesthesia of lower limbs. But he did not develop a definite sensory level at anytime during the course of his illness.

**Figure 1:**
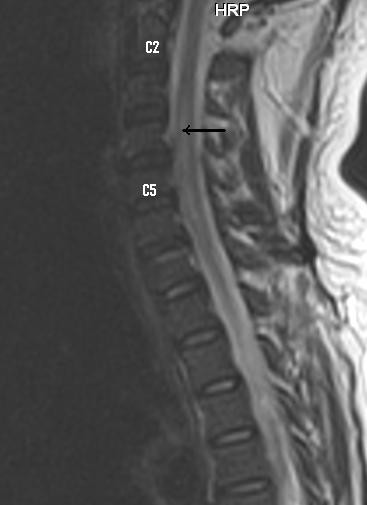
T2 weighted image showing hyperintensity of the central portion of the mid cervical cord (arrow).

**Figure 2:**
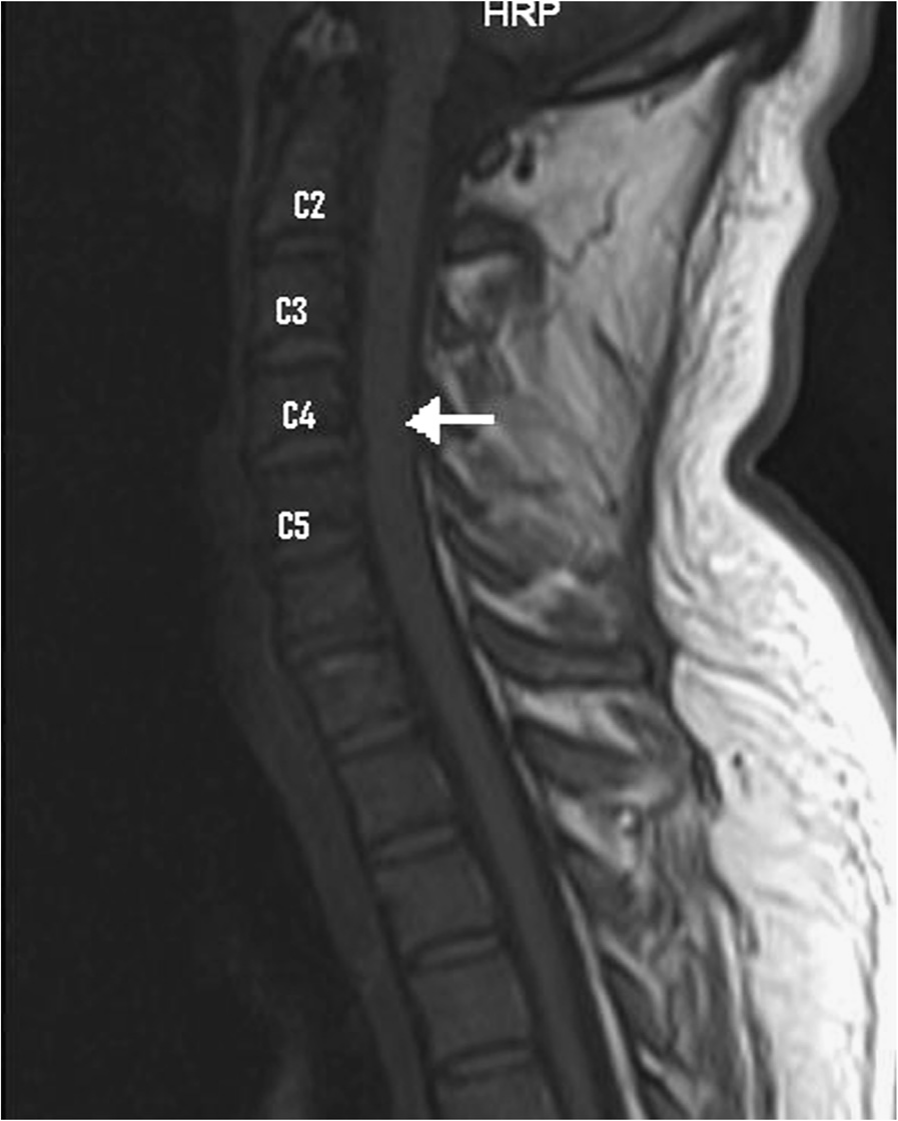
T1 weighted image showing mild swelling of mid cervical cord (arrow).

A nerve conduction study failed to show demyelination or axonal loss. Magnetic resonance imaging study of the spine showed hyperintensity of central portion of mid cervical cord from C 2 to C 5 level in T2 weighted and FLAIR images (Figure 1) There was mild swelling of cervical cord without significant change of signal intensity on T1 weighted image (Figure 2).

Basic investigations were as follows; Na^+^ 139 mmol/l, K^+^-4.2 mmol/l, Serum creatinine-62 μmol/l, blood urea 8.7 mmol/l, White cell count 5000/mm^3^(N-76,L-18), platelet count 17500/mm^3^. Haemoglobin 9.3 g/dl, AST-51u/l, ALT-74u/l, Bilirubin 12 μmol/l, ESR-12 mm/hr, CRP-0.4 mg/dl, VDRL-negative, Fasting blood sugar 5.4 mmol/l.

Cerebrospinal fluid analysis done on the tenth day after onset of weakness was suggestive of a viral CNS infection with a protein content of 300 mg/l, glcose 4.3 mmol/l(RBS 10.9 mmol/l) and a leukocyte count of 350/mm^3^(92% lymphocytes). CSF infectious screen was positive for cytomegalovirus PCR(sensitivity more than 100copies/ml). This test was repeated and result was confirmed. Cytomegalovirus IgG and IgM were not detected in CSF. Polymerase chain reaction of CSF for herpes simplex virus(HSV) and Japanese encephalitis virus(JEV) were negative. Oligoclonal bands were not detected in CSF.

Considering the overall clinical picture, a working diagnosis of CMV associated transverse myelitis was made. Immunoglobulin levels and lymphocyte subset analysis failed to identify any immunodeficiency while HIV serology was negative.

Patient received treatment with intravenous ganciclovir for a duration of 21 days starting on the 10th day of illness. He was also given maximal supportive care with physiotherapy and psychological support. A second CSF analysis done 22 days after the onset of symptoms revealed no cells and protein content of 390 mg/l. (CSF glucose 3.6 mmol/l RBS-6.5 mmol/l) Repeated CMV serology in blood, CSF and CSF PCR for CMV were negative on the 22nd day of illness.

One month after the onset of symptoms the upper limb power was improved (grade 5) but the lower limb power remained grade 3. He was transferred to a long term rehabilitation facility.

## Conclusions

The findings in our patient are suggestive of acute transverse myelitis involving spinal segments C3 to C5. According to the proposed diagnosis criteria for Transverse myelitis [3] our patient had evidence of spinal cord inflammation evidenced by CSF pleocytosis and MRI appearance. Although a clear sensory level was absent, he had sensory, motor and autonomic dysfunction attributable to the spinal cord, bilateral involvement, progression to nadir between 4 hours to 21 days after symptom onset and absence of compressive lesions.

Patient’s clinical history was compatible with acute cytomegalovirus infection. Polymerase chain reaction has been found to be the most reliable method for the CMV related CNS infections [6]. While cytomegalovirus is an identified cause of myelitis in immunocompromised hosts, it is rare in the immunocompetent people with only few published case reports (Table 1). Cytomegalovirus PCR has demonstrated the presence of CMV DNA in blood in only two reported cases of CMV associated transverse myelitis in the immunocompetent patients [7,8]. In other immunocompetent patients with CMV transverse myelitis, either PCR was not done or was negative. All ten patients had positive serology for CMV antibody while in this patient serology was negative.

Five patients had their CSF tested for CMV DNA by PCR and all were negative. Therefore our patient is the first reported case of CMV DNA positivity in CSF among all immunocompetent patients with CMV transverse myelitis. Absence of CMV DNA in CSF in previous cases has been considered to favour immune mediated rather than direct virus induced injury to the cord [5,6]. The presence of CMV DNA in CSF while serology remained negative may indicate direct virus induced injury has to be considered in the pathogenesis of CMV transverse myelitis. The reason for negativity of CMV antibodies in our patient could be either due to low sensitivity of the serological test or early treatment leading to destruction of virus before an immunological response is mounted.

Out of the ten patients MRI findings were abnormal in five patients, while others either had normal MRI appearances or MRI was not performed. Commonest MRI finding was signal hyperintensity in T2 sequences. Other documented MRI findings include T1 hypointensity as well as hyper intensity. Contrast enhancement of the spinal cord was documented only in a single case report [5].

This case emphasizes the fact that CMV should be considered as an aetiological agent in patients presenting with a clinical syndrome suggestive of transverse myelitis even though they are immunocompetent. Cytomegalovirus DNA detection in CSF and MRI appearance can help in the diagnosis of CMV transverse myelitis. Although randomised trials are lacking, ganciclovir can be useful in the management of CMV transverse myelitis.

## Consent

Written informed consent was obtained from the patient for publication of this Case report and accompanying images. A copy of the written consent is available for review by the Series Editor of this journal.

## Competing interests

The authors declare that they have no competing interests.

## Authors’ contributions

All four authors were involved in the clinical management of the patient. SK prepared the manuscript. DG and YU provided assistance in literature survey and manuscript preparation. HF critically analysed the final manuscript with regard to scientific data. All four authors read and approved the final manuscript.

## Authors’ information

SK is a registrar in medicine attached to the national hospital of Sri Lanka, which is the final tertiary care referral centre in the island. DG and YU are senior registrars in medicine attached to the same insitute. HF is a consultant physician atttached to the national hospital of Sri Lanka and the clinical trainer of the other three authors.

## References

[B1] Rafailidis PI Mourtzoukou EG Varbobitis IC Falagas ME Severe cytomegalovirus infection in apparently immunocompetent patients: a systematic review Virol J 2008 5 47 10.1186/1743-422X-5-47 18371229 PMC2289809

[B2] Griffiths P Cytomegalovirus infection of the central nervous system Herpes 2004 11 Suppl 2 95A 104A 15319096

[B3] Transverse Myelitis Consortium Working Group Proposed diagnostic criteria and nosology of acute transverse myelitis Neurology 2002 59 499 505 12236201 10.1212/wnl.59.4.499

[B4] Ben Abdelhafidh N Battikh R Laabidi J M'sadek F Ajili F Ben Moussa M Amor A Ben Abdallah N Louzir B Othmani S [Cytomegalovirus myelitis in immunocompetent adult] Rev Med Interne 2006 27 883 885 10.1016/j.revmed.2006.04.020 16797108

[B5] Rigamonti A Usai S Ciusani E Bussone G Atypical transverse myelitis due to cytomegalovirus in an immunocompetent patient Neurol Sci 2005 26 5 351 354 10.1007/s10072-005-0506-6 16388372

[B6] Fux CA Pfister S Nohl F Zimmerli S Cytomegalovirus-associated acute transverse myelitis in immunocompetent adults Clin Microbiol Infect 2003 9 1187 1190 10.1111/j.1469-0691.2003.00796.x 14686983

[B7] Karacostas D Christodoulou C Drevelengas A Paschalidou M Ioannides P Constantinou A Milonas I Cytomegalovirus-associated transverse myelitis in a non-immunocompromised patient Spinal Cord 2002 40 145 149 10.1038/sj.sc.3101265 11859442

[B8] Giobbia M Carniato A Scotton PG Marchiori GC Vaglia A Cytomegalovirus-associated transverse myelitis in a non-immunocompromised patient Infection 1999 27 228 230 10.1007/BF02561538 10378139

[B9] Baig SM Khan MA Cytomegalovirus-associated transverse myelitis in a non-immunocompromised patient J Neurol Sci 1995 134 210 211 10.1016/0022-510X(95)00270-X 8747869

[B10] Miles C Hoffman W Lai CW Freeman JW Cytomegalovirus-associated transverse myelitis Neurology 1993 43 10 2143 2145 8413984 10.1212/wnl.43.10.2143-a

[B11] Tobita M Komiyama A Naito M Johkura K Hasegawa O Cytomegalovirus ascending myelitis in an immunocompetent adult Rinsho Shinkeigaku 1993 33 915 917 8261709

[B12] Tyler KL Gross RA Cascino GD Unusual viral causes of transverse myelitis: hepatitis A virus and cytomegalovirus Neurology 1986 36 855 858 10.1212/WNL.36.6.855 3010183

[B13] Kabins S Keller R Naragi S Peitchel R Viral ascending radiculomyelitis with severe hypoglycorrhachia Arch Intern Med 1976 136 933 935 10.1001/archinte.1976.03630080067020 182099

[B14] Eddleston M Peacock S Juniper M Warrell DA Severe Cytomegalovirus Infection in Immunocompetent Patients Clin Infect Dis 1997 24 52 56 10.1093/clinids/24.1.52 8994755

